# Secretagogin is expressed in sensory CGRP neurons and in spinal cord of mouse and complements other calcium-binding proteins, with a note on rat and human

**DOI:** 10.1186/1744-8069-8-80

**Published:** 2012-10-29

**Authors:** Tie-Jun Sten Shi, Qiong Xiang, Ming-Dong Zhang, Giuseppe Tortoriello, Henrik Hammarberg, Jan Mulder, Kaj Fried, Ludwig Wagner, Anna Josephson, Mathias Uhlén, Tibor Harkany, Tomas Hökfelt

**Affiliations:** 1 School of Life Science and Technology, Harbin Institute of Technology, 150001, Harbin, China; 2 Department of Neuroscience, Karolinska Institutet, Stockholm, Sweden; 3 Department of Dental Medicine, Karolinska Institutet, Stockholm, Sweden; 4 Division of Molecular Neurobiology, Department of Medical Biochemistry & Biophysics, Karolinska Institutet, Stockholm, Sweden; 5 Department of Clinical Science and Education, Södersjukhuset (The Southern Hospital), Karolinska Institutet, Stockholm, Sweden; 6 Department of Neuroscience, Science for Life Laboratory, Karolinska Institutet, Stockholm, Sweden; 7 Department of Medicine III, Medical University of Vienna, Vienna, Austria; 8 Science for Life Laboratory, Albanova University Center, Royal Institute of Technology (KTH), Stockholm, Sweden; 9 European Neuroscience Institute, University of Aberdeen, Aberdeen, UK

**Keywords:** Calbindin D-28k, Calretinin, Dorsal horn, Dorsal root ganglion, Nerve injury, Parvalbumin, Trigeminal ganglion

## Abstract

**Background:**

Secretagogin (Scgn), a member of the EF-hand calcium-binding protein (CaBP) superfamily, has recently been found in subsets of developing and adult neurons. Here, we have analyzed the expression of Scgn in dorsal root ganglia (DRGs) and trigeminal ganglia (TGs), and in spinal cord of mouse at the mRNA and protein levels, and in comparison to the well-known CaBPs, calbindin D-28k, parvalbumin and calretinin. Rat DRGs, TGs and spinal cord, as well as human DRGs and spinal cord were used to reveal phylogenetic variations.

**Results:**

We found Scgn mRNA expressed in mouse and human DRGs and in mouse ventral spinal cord. Our immunohistochemical data showed a complementary distribution of Scgn and the three CaBPs in mouse DRG neurons and spinal cord. Scgn was expressed in ~7% of all mouse DRG neuron profiles, mainly small ones and almost exclusively co-localized with calcitonin gene-related peptide (CGRP). This co-localization was also seen in human, but not in rat DRGs. Scgn could be detected in the mouse sciatic nerve and accumulated proximal to its constriction. In mouse spinal cord, Scgn-positive neuronal cell bodies and fibers were found in gray matter, especially in the dorsal horn, with particularly high concentrations of fibers in the superficial laminae, as well as in cell bodies in inner lamina II and in some other laminae. A dense Scgn-positive fiber network and some small cell bodies were also found in the superficial dorsal horn of humans. In the ventral horn, a small number of neurons were Scgn-positive in mouse but not rat, confirming mRNA distribution. Both in mouse and rat, a subset of TG neurons contained Scgn. Dorsal rhizotomy strongly reduced Scgn fiber staining in the dorsal horn. Peripheral axotomy did not clearly affect Scgn expression in DRGs, dorsal horn or ventral horn neurons in mouse.

**Conclusions:**

Scgn is a CaBP expressed in a subpopulation of nociceptive DRG neurons and their processes in the dorsal horn of mouse, human and rat, the former two co-expressing CGRP, as well as in dorsal horn neurons in all three species. Functional implications of these findings include the cellular refinement of sensory information, in particular during the processing of pain.

## Background

Calcium-binding proteins (CaBPs) play a major role in neuronal functions, and their cellular distribution in the nervous system has in many cases been thoroughly mapped by immunohistochemistry [1,2]. In particular, parvalbumin (PV), calretinin (CR) and calbindin D-28k (CB) have received much attention due to their robust, developmentally regulated and cell type-specific expression in the nervous system, and have emerged as effective markers to identify subpopulations of neurons [2-4]. In general terms, these proteins act either as Ca^2+^ sensors or buffers of Ca^2+^ transients in neurons, defined by their molecular properties and the signaling context they participate in [5]. Chemically, CaBPs are characterized by a tandem repeat of the Ca^2+^-binding loop surrounded by two helices, the EF-hand binding site [1,6,7].

Secretagogin (Scgn) is a recently cloned member of the EF-hand CaBP superfamily, first identified from a human pancreatic cDNA library by immunoscreening with the murine monoclonal antibody D24 generated using human insulinoma as immunogen [8,9]. Structurally, Scgn’s deduced amino acid sequence specifies a protein of 276 amino acids with a calculated molecular mass of 32 kDa that can bind up to four Ca^2+^ ions simultaneously [8]. Using immunohistochemical methodology, Scgn has been detected in several tissues, such as the brain of various mammalian species including humans [10-16], where it may associate with SNAP-25 [17], a protein(s) participating in the vesicular exocytosis of neurotransmitters [18], possibly neurodegeneration [10-12], as well as development, including embryonic expression in dorsal root ganglia (DRGs) and trigeminal ganglia (TGs) [15].

DRGs are composed of a considerable number of neuronal subtypes underpinning specific sensory modalities, and all originating from a common pool of embryonic precursors [19,20]. As in other neuronal systems (see above), CaBPs have been used as markers in numerous studies reporting the distribution of CaBPs in sensory ganglia at different levels. In particular, DRGs and TGs have been extensively analyzed. In addition, the spinal dorsal horn and spinal trigeminal nucleus, regions important for sensory information processing, including pain, have been studied. They include chicken [21-23], Xenopus laevis [24], turtle [25], zebra fish [26], dog [27], mouse [28-32] and, most thoroughly, rat [2,21,33-50]. However, caveats of knowledge exist regarding the cellular sites of Scgn mRNA expression and protein distribution in DRGs or spinal cord.

In the present study we have therefore analyzed, with quantitative (real-time) PCR (qPCR), *in situ* hybridization and high-resolution immunohistochemistry, the localization of Scgn in mouse DRGs (mDRGs) and spinal cord, with emphasis on its possible co-localization with PV, CR, and CB, as well as with calcitonin gene-related peptide (CGRP) or isolectin B4 (IB4), classic markers of nociceptive neurons [51,52]. In addition, the presence of three further molecules known to be expressed in DRGs/dorsal horn was studied: transient receptor potential vanilloid subtype 1 (TRPV1) [53], gastrin releasing peptide (GRP) [54-56], and protein kinase C gamma (PKCgamma) [57]. Dorsal root transection and unilateral peripheral sciatic nerve injury were performed in mice. Finally, we have, in a preliminary way, studied the extent of phylogenetic conservation in Scgn’s distribution by comparing mouse DRGs, TGs and spinal cord with rat DRGs (rDRGs), rat TGs (rTGs) and rat spinal cord, as well as with human DRGs (hDRGs) and spinal cord. Some of these results were presented in a preliminary form at the 13^th^ World Congress on Pain [58].

## Results

### Scgn mRNA detection: methodological considerations and tissue distribution pattern

Recently, Scgn has been localized in the brain with immunohistochemistry using affinity-purified antibodies raised against distinct peptide domains (“epitopes”) of this protein [15], producing results that correspond well with publicly-available mRNA distribution maps [59]. Nevertheless, correlative analysis of Scgn mRNA and protein has not been performed. Therefore, we first probed Scgn mRNA distribution in the olfactory bulb, containing highest Scgn protein and mRNA levels in the nervous system [15,16]. We visualized, using riboprobes, Scgn mRNA as a “band” in deep neurons populating the granular layer (Additional file 1: Figure S1A), as well as the inner sublayer of the external plexiform layer (Additional file 1: Figure S1A_1_). In addition, Scgn mRNA, though at relatively low levels, was found in cells scattered around olfactory glomeruli Additional file 1: Figure S1A_2_,B), likely periglomerular interneurons. Thus, corresponding Scgn mRNA (Additional file 1: Figure S1A-A_2_) and protein distribution patterns (Additional file 1: Figure S1C,C_1_) support the specificity of the antibodies used in the present and previous [15,16,60] studies.

**Figure 1:**
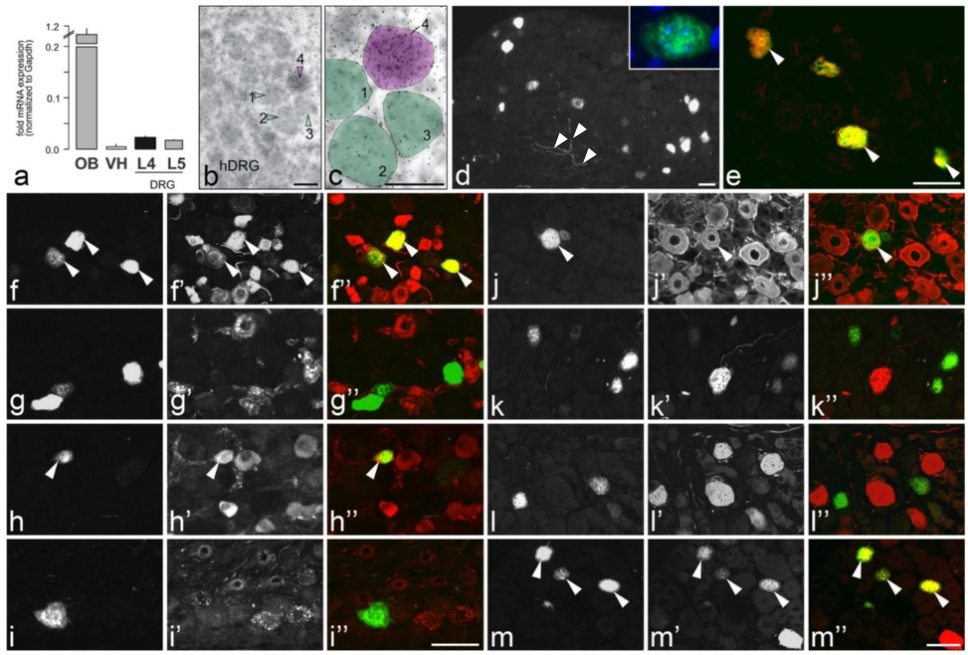
**Scgn expression in DRGs.** (**a**) Quantitative (real-time) PCR detection of Scgn mRNA in mouse olfactory bulb (OB), ventral and dorsal horns (VH/DH) of L4-5 spinal cord segments, and L4-5 DRGs. Scgn mRNA is found expressed at significantly lower levels in VH and DRGs than in the OB. Scgn mRNA is under the detection threshold in DH. Gapdh is used as internal control. (**b-c**) Scgn mRNA expression in human DRG (hDRG). Semitransparent purple and green shades outline the surface area of Scgn mRNA-positive and negative cells, respectively. Note that tissues are counterstained by cresyl violet after emulsion-radiography of the mRNA hybridization signal. Scgn-LI in control L5 mDRGs (**d-m**”). Immunofluorescence micrographs of sections incubated with antiserum to Scgn (**d-m**), CGRP (**f’**), IB4 (**g’**), TRPV1 (**h’**), GRP (**i’**), NF200 (**j’**), CB (**k’**), PV (**l’**) or CR (**m’**). Color micrographs show merged micrographs after double-staining (**f-f”**, **g-g”**, **h-h”**, **i-i”**, **j-j”**, **k-k”**, **l-l”**, **m-m”** show, respectively, the same section). (**d**) Several Scgn-IR cells are seen. Boxed area shows Scgn staining also in the nucleus. Arrowheads indicate Scgn-positive fibers. (**e**) Double labeling shows the same staining pattern present after incubation with two different Scgn antibodies (arrowheads). (**f-m”**) Arrowheads indicate coexistence of Scgn (yellow) with CGRP (**f-f”**), TRPV1 (**h-h”**), NF200 (**j-j”**) and CR (**m-m”**), most pronounced for Scgn plus CGRP (**f-f”**). Scgn-LI cannot be seen in IB4-positive (**g-g”**), GRP (**i-I”**), CB-(**k-k”**) or PV-IR (**l-l”**) neurons. Scale bars indicate 10 μm (**b**,**c**), 50 μm (**d,e-m”**).

Next, we profiled relative Scgn mRNA levels between amongst mouse olfactory bulb, dorsal and ventral spinal cord, and DRGs lumbar 4 and 5 (L4-5) by means of qPCR. While Scgn mRNA levels in the olfactory bulb were exceptionally high (Figure 1a), they were under detection threshold and at very low levels in the dorsal and ventral spinal horns, respectively (Figure 1a). Moderate Scgn mRNA levels were detected in DRGs (Figure 1a, *n* = 3/region) suggesting that only a restricted population of DRG neurons might be Scgn immunoreactive (IR).

We also addressed Scgn expression in DRGs using *in situ* hybridization. We demonstrate, by using oligoprobes, that a small population of cells in the hDRG expresses notable levels of Scgn mRNA (Figure 1b,c). However, we could not reliably detect *in situ* hybridization signal in mDRGs, probably reflecting the sparse mRNA levels found in our qPCR analysis.

### Expression of Scgn protein in neurons in DRGs

In adult mDRGs, 7.3±1.2% of all neuron profiles (NPs) were Scgn-IR (Figure 1d), including nuclear-like Scgn distribution in some mDRG neurons (Figure 1d and inset). Only few and weakly fluorescent processes were detected in the DRGs (Figure 1d). Most Scgn-IR NPs (73%) were small-sized (Figure 1d). The same staining pattern was observed after incubation with a rabbit anti-human Scgn antibody raised against a different epitope revealing double-labeled (identical) cell bodies (Figure 1e). In mDRGs, Scgn-IR neurons abundantly expressed CGRP (98.0±3.4%) (Figure 1f-f"), a phenotypic marker for peptidergic neurons, whereas none of the Scgn-IR neuron co-localized with IB4 (Figure 1g-g"), a marker for non-peptidergic neurons. TRPV1-like immunoreactivity (LI) was found in 20.0±2.5% of all Scgn-IR NPs (Figure 1h-h"). In contrast, Scgn- and GRP-LIs did not co-exist (Figure 1i-i´´). Very few of the Scgn-IR neurons in mouse expressed NF200-LI (Figure 1j-j´´).

Scgn expression in mDRGs was compared with those of the three “classical” CaBPs using double-label immunohistochemistry (that is PV, CB and CR). Scgn revealed a separate neuron population, only occasionally expressed in NPs immunoreactive for CB (0.5±0.5%; Figure 1k-k´´), PV (0.4±0.4%; Figure 1l-l´´), although some expressed CR (2.1±1.3%; Figure 1m-m´´).

**Figure 2:**
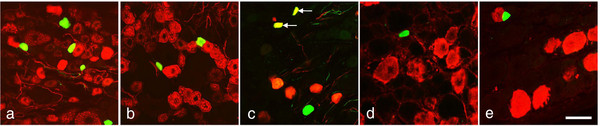
**Scgn-LI in control L5 rDRGs.** (**a-e**) Double-immunofluorescence micrographs of sections incubated with antiserum to Scgn (**a-e**; green) plus CGRP (**a**; red), IB4 (**b**; red), CB (**c**; red), PV (**d**; red) or CR (**e**; red). Arrows indicate coexistence of Scgn with CB (**c**; yellow). Scale bar indicates 50 μm (**a-e**).

In control rDRGs, 3.0±0.7% of all NPs were Scgn-IR, mainly small-sized (Figure 2a-e). None of the Scgn-IR neurons co-expressed CGRP (Figure 2a), contrasting mouse, or IB4 (Figure 2b). With regard to coexistence with the three CaBPs, 42.5±2.2% of the Scgn-IR NPs were CB-IR (Figure 2c). Neither PV nor CR-IR NPs were Scgn-positive (Figure 2d,e).

**Figure 3:**
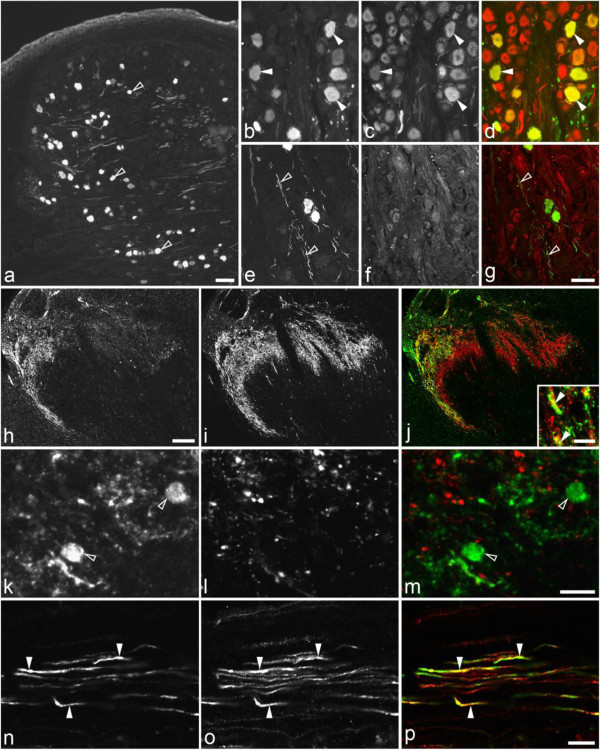
**Scgn-LI in hDRG and human spinal cord.** (**a-d**) Immunofluorescence micrographs of section incubated with antiserum against Scgn (**a**,**b**,**d**) or/and CGRP (**c**,**d**). (**b-d**) show same section processed for double-immunofluorescence. Arrowheads indicate Scgn-IR neurons (**a**), and neurons co-expressing (solid arrowheads in **d**; yellow) Scgn- (**b**, **d**) and CGRP-LI (**c**, **d**). Note that all Scgn-IR neurons are CGRP-IR. (**e-g**) Immunofluorescence micrographs of section incubated with antiserum against Scgn (**e**, **g**) or/and IB4 (**f**, **g**). **e-g** show same section processed for double-immunofluorescence. Arrowheads indicate Scgn-IR fibers (**e**, **g**). Note that no Scgn-IR neuron is IB4-positive. (**h-p**) Immunofluorescence micrographs of a section incubated with antiserum against Scgn (**h**,**j**,**k**,**m**,**n** and **p**) or/and CGRP (**i**,**j**,**l**,**m**,**o** and **p**). **h-j** show the double-labeling of Scgn and CGRP in human spinal cord at low magnification. Scgn-IR fibers are concentrated in the lateral superficial layer and some of them show co-localization with CGRP as indicated by arrowheads (inset in **j**). **k-m** show Scgn-IR interneurons surrounded by some positive nerve endings in inner layer of lamina II (arrowheads indicate Scgn-IR interneurons). **n-p** show the co-localization of Scgn-LI and CGRP-LI in axons in the dorsal roots (arrowheads, yellow). Scale bars indicate 200 μm (**a**, **h-j**), 100 μm (**b-g**), 20 μm (**k**-**m**, **n-p** and inset in **j**).

In the hDRGs, 13.3±0.4% of all NPs were Scgn-IR (Figure 3a), and there were also distinct processes (Figure 3e,g). Of the Scgn-IR NPs, 94.1±3.6% were CGRP-IR (Figure 3b-d), but no IB4-positive ones were detected (Figure 3e-g). 12.3±3.8% of CGRP-IR NPs expressed Scgn (Figure 3b-d).

**Figure 4:**
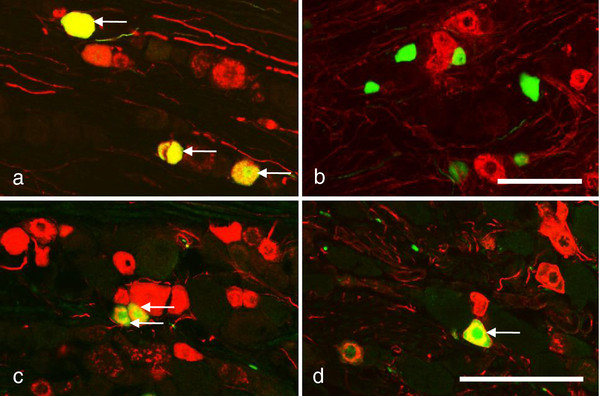
**Scgn-LI in mTG and rTG.** (**a-d**) Immunofluorescence micrographs showing mTG (**a**,**b**) and rTG (**c**,**d**) after double-staining with antiserum against Scgn (**a-d**; green) and CGRP (**a**,**c**; red) or IB4 (**b**,**d**; red). Arrows indicate coexistence of Scgn (yellow) with CGRP (a,c) or IB4 (d). Note coexistence of Scgn with CGRP in m- and rTGs, and of Scgn with IB4 in rTG but not in mTG. Scale bars indicate 50 μm (**a=b**; **c=d**).

In mTGs 5.9±0.5% NPs were Scgn-positive, and 91.7±1.9% of Scgn-IR NPs coexpressed CGRP (Figure 4a), whereas no Scgn-positive NPs expressed IB4 (Figure 4b). In rTGs, 80.5±7.9% and 75.3±9.5% of Scgn-IR NPs were CGRP- and IB4-IR, respectively (Figure 4c,d).

### Localization of Scgn protein in spinal cord and sciatic nerve

**Figure 5:**
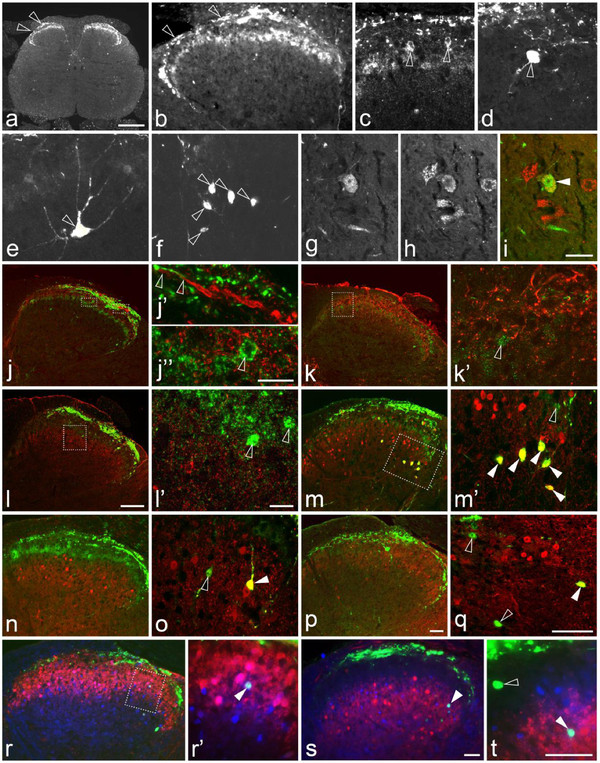
**Scgn-LI in control mouse lumbar spinal cord.** (a-t) Immunofluorescence micrographs of sections incubated with antiserum against Scgn (**a-g**, **i-t**), CGRP (**h**, **i**), TRPV1 (**j-j”**), GRP (**k, k’**), PKC gamma (**l**, **l’**), CB (**m**, **m’**, **r-t**), PV (**n**, **o**), or/and CR (**p**, **q**). Scgn-IR fibers are seen in the superficial dorsal horn (arrowheads in **a**, **b**) and also processes in lamina III (**a**, **b**). Scgn-IR cell bodies are present in lamina II (small-sized in **c**), a few in III (**d**) or medial (multi-polar in **e**) and lateral (small-sized in **f**) parts of lamina IV. (**g-i**) Ventral horn neurons express Scgn-LI (**g**, **i**) and are mostly CGRP-IR (arrowhead in **i**). Scgn-IR (**j”**, **k’**, **I’**; green) neurons do not express TRPV1 (**j,j”**), GRP (**k**, **k’**) or PKC gamma (**l**, **l’**) in dorsal horn. (**m-q**) Filled Arrowheads indicate coexistence (yellow) of Scgn-LI (**m’**, **o**, **q**, green) and CB (**m**, red), PV (**o**; red) or CR (**q**; red) in the dorsal horn. (**r-t**) Triple-labeling of superficial dorsal horn showing (**r** and **r’** same section) a local neuron (light blue; filled arrowhead) coexpressing Scgn (**r’**; green) and CB (**r’**; read), but not PV (**r’**; dark blue). **s** and **t** show neurons (filled arrowhead; light blue) containing Scgn (green) and CB (red), but not CR (dark blue). Empty arrowheads indicate a dorsal horn neuron only express Scgn-LI (j'',k',l',o,q,t) or a Scgn-IR fiber (j'; green) does not overlap with TRPV1-IR fiber (j'; red). Scale bars indicate 200μm (**a**), 50 μm (**b=m=n=p**; **c=d=e=f**; **g=h=i**; **j=k=l**; **j’=j”**; **k’=l’**; **m’=o=q**; **r=s**; **r’=t**).

**Figure 6:**
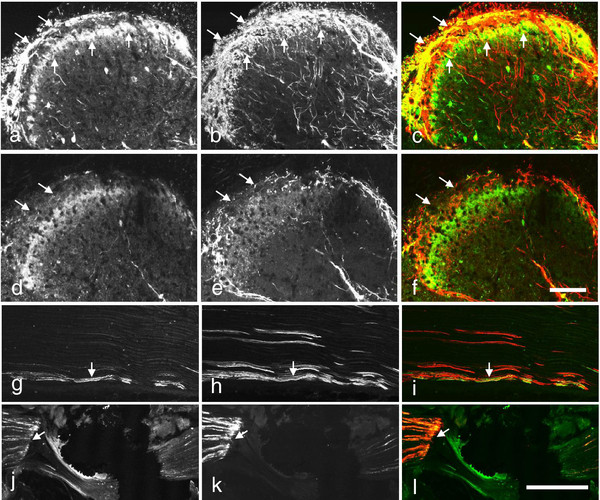
**Scgn-LI in mouse spinal cord and sciatic nerve after rhizotomy or nerve ligation.** (**a-f**) Immunofluorescence micrographs of sections incubated with antiserum against Scgn- (**a**,**c**,**d**,**f**) and CGRP- (**b**,**c**,**e**,**f**) LIs in lumbar spinal cord 10 days after unilateral, dorsal rhizotomy (**d-f**). (**a-f**) On the contralateral side both Scgn- and CGRP-LIs are strong in the superficial dorsal horn, the former displaying two bands (arrows in **a**, **c**), the latter filling out layers I and outer II (arrows in **b**,**c**). The merged micrograph (**c**) shows that the deeper band of Scgn-LI, consisting mainly of cell bodies, essentially remains in inner lamina II, without much overlap with the CGRP-LI (**d-f**). There is a strong ipsilateral decrease of both Scgn-LI (cf. **d**, **f** with **a**, **c**; arrows) and CGRP-LI (cf. **e**, **f** with **b**, **c**), compared to a contralateral side (**a-c**). (**g-i**) Scgn-LI is weekly (arrows in **g**, **i**), and CGRP more extensively (arrow in **h**, **i**) expressed in the control sciatic nerve, and both accumulate, on the proximal side of a-10-hour ligation (arrows in **j**, **k**). Double-staining shown in merged color micrographs indicates possible coexistence (arrows in **i**, **l**). Scale bars indicate 100 μm (**a-f**; **g-l**).

Scgn-LI was found both in the neuronal cell bodies and fibers/processes in the mouse spinal cord: Scgn-IR fibers formed a dense plexus in the superficial dorsal horn (Lissauer’s tract) (Figures 5a,b; 6a). Scgn-IR neurons were mainly found in inner lamina II (Figures. 5c; 6a), but some cells were also seen in layers II-V, including both small neurons (Figure 5f) and large, multipolar neurons (Figure 5d,e). In the ventral horn, Scgn-positive neurons were sporadically seen (Figure 5g), and some of them (3.0%) co-expressed CGRP (Figure 5g-i), in this region a marker for motoneurons [61]. 

In mouse dorsal horn, using double staining, we detected most superficial Scgn-IR fibers were CGRP-IR (Figure 6a-c), however, the CGRP-IR fibers extended deeper than the Scgn-positive ones (Figure 6a-c). The Scgn-positive interneurons and fibers lacked TRPV1-LI (Figure 5j-j´´). None of the Scgn-positive fibers or neuronal cell bodies were GRP-IR (Figure 5k,k´), nor were Scgn-positive neuronal cell bodies in lamina II PKCgamma-positive (Figure 5l,l´). Scgn-LI was present in some CB-IR neurons (Figure 5m,m´), in very few PV-IR (Figure 5n,o) and CR-IR neurons (Figure 5p,q). The neuron in Figure 5r and r´ expressing Scgn and CB appeared PV-negative. There were also neurons co-expressing Scgn and CB but apparently not CR (Figure 5s,t).

Unilateral dorsal rhizotomy strongly reduced both Scgn- and CGRP-LIs in the superficial region of the ipsilateral dorsal horn as compared to the contralateral side (cf. Figure 6a-c with d-f). However, there were still cell bodies and processes in inner lamina II (Figure 6d,f). In the sciatic nerve, a moderate number of Scgn-IR axons could be seen (Figure 6g), however fewer than the CGRP-positive ones (Figure 6h), partially overlapping (Figure 6i). Ten hours after ligation of the sciatic nerve there was distinct accumulation of Scgn-LI (Figure 6j) and CGRP-LI (Figure 6k) proximal to the site of the injury. In contrast to the normal nerve, there seemed to be a more equal number of fibers immunoreactive for Scgn and CGRP with prominent overlap, further supporting their co-existence (Figure 6l).

**Figure 7:**
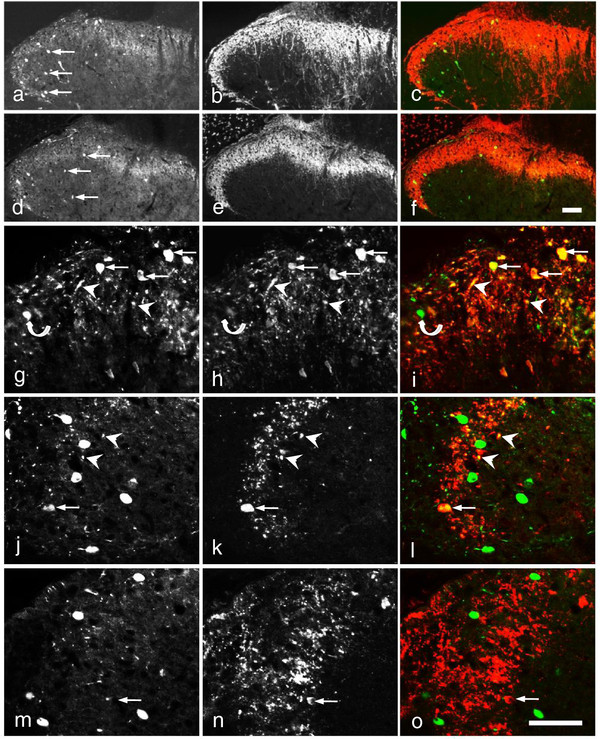
**Scgn -LI in control rat spinal dorsal horn.** (**a-f**) Immunofluorescence micrographs after double-staining with antiserum against Scgn (**a,c,d,f**), CGRP (**b,c**) or IB4 (**e**,**f**). (**a-f**) Scgn-IR neurons (arrows) are seen in the dorsal horn, both in superficial and deeper layers (**a**,**d**). They partly overlap with CGRP-LI (**b**) and IB4 staining (**e**), as seen in merged color micrographs (**c,f**). (**g-o**) Double-immunofluorescence micrographs of sections incubated with Scgn antiserum (**g**,**i**,**j**,**l**,**m**,**o**; green) plus CB (**h**, **i**; red), PV (**k**,**l**; red) or CR antiserum (**n**,**o**; red). Coexistence is often seen for CB in cell bodies (arrows in **g-i**) and processes (arrowheads in **g-i**), less so for PV (arrow/arrowheads in **j-l**) and, here, none for CR (**m-o**). Arrows indicate coexistence (yellow) of Scgn-LI (**i**, **l**) and CB (**i**) and PV (**l**) in the dorsal horn neurons. Arrowheads indicate coexistence of Scgn-IR fibers (**i**,**l**) and CB (**i**) or PV (**l**). Curved arrows indicate a Scgn-positive neuron that does not express CB (**g-i**). Arrows indicate a CR-positive but Scgn negative neuron (**m-o**). Bar in f indicates 100 μm (**a-f**) and 50 μm (**g-o**).

In the rat dorsal horn, Scgn-LI was less prominent and mainly observed in medial, inner lamina II (Figure 7a,d), partly overlapping with CGRP-IR fibers (Figure 7b,c) and with IB4 (Figure 7e,f) stainings. There was a moderate number of small Scgn-IR cell bodies in laminae I-IV (Figure 7a,d). Double-staining experiments showed that, in the rat superficial dorsal horn, some Scgn-IR neurons expressed CB- (Figure 7g-i) or PV- (Figure 7j-l), but not CR (Figure 7m-o). In contrast to mouse, Scgn-LI was not found in rat ventral horn neurons (data not shown).

In the human spinal cord, a dense network of Scgn-IR fibers was observed in the superficial, especially lateral, dorsal horn, most of which were CGRP-IR (Figure 3h-j). As in mouse, the CGRP fibers extended ventrally beyond the Scgn zone. A few Scgn-positive cell bodies, surrounded by Scgn nerve endings were seen in inner lamina II, but they did not, as in mouse, from a distinct band (Figure 3k-m). Scgn-IR/CGRP-IR axons were also observed in accidentally included dorsal roots (Figure 3n-p).

### Scgn protein expression after peripheral nerve injury

**Figure 8:**
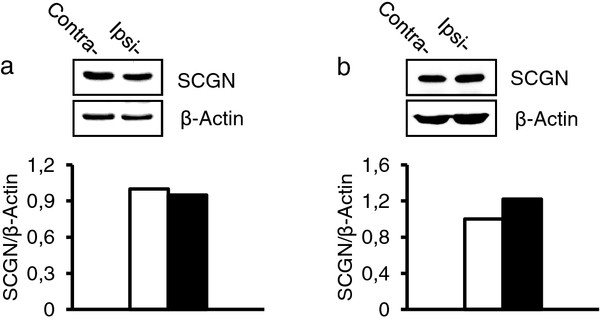
**Expression of Scgn protein after sciatic nerve axotomy as shown with western blot.** (**a**, **b**) L4-5 DRGs (**a**) and L 4–5 spinal cord segments (**b**) after unilateral nerve transection using antiserum against Scgn and β-Actin. No obvious changes in Scgn expression are seen in ipsi- and contralateral DRGs (**a**) or spinal cord (**b**), respectively. Quantification of three western blots shows similar levels of Scgn between ipsi- and contralateral DRGs or spinal cord.

Transection of the sciatic nerve did not significantly affect the percentage of Scgn-IR NPs in ipsilateral mouse DRGs as compared to contralateral ones (6.6±1.0% vs. 6.3±0.9; P>0.05). In agreement, Western blotting showed no change of Scgn protein levels in ipsilateral vs. contralateral mDRGs (Figure 8a). Finally, in mouse spinal cord, the total protein levels of Scgn did not change after peripheral nerve injury (Figure 8b).

## Discussion

The present study shows that Scgn, a recently identified member of the CaBP superfamily [8], is expressed in distinct neuronal populations at the spinal level of several species. In mDRGs, subpopulations of these nociceptive neurons express CGRP (98%)- and TRPV1-(~20%) but are IB4-negative [51,52]. Thus, the TRPV1-IR population of these neurons may be sensitive to noxious heat [53]. The apparent lack of GRP in Scgn-IR DRG neurons indicates that they are not involved in itching [56], and Scgn-positive, PKCgamma-negative dorsal horn interneurons may not be excitatory [57]. We have, however, not been able to assign these neurons to any of the categories identified in extensive developmental studies [19,20]. They are distinctly different from those harboring the three most-studied CaBPs (PV, CB and CR).

Scgn mRNA was detected in a subpopulation of hDRG neurons, and Scgn mRNA transcripts were found in mDRGs by means of qPCR. The specificity of the antiserum has further been supported by adsorption experiments and Western blot analysis, as also shown in previous studies on brain [15,16], where results were compared with those in the Allen brain atlas [59]. Finally, double-staining experiments with two different antisera raised against different epitopes and in two animal species stained the same cell bodies in mDRGs.

### Scgn is present in all major compartments of the mDRG neuron

An interesting question is to what extent Scgn produced in the mDRGs is transported to the dorsal horn. Our findings with dorsal rhizotomy suggest that the staining in the superficial layers, but not in inner lamina II, originates in the DRGs. Moreover, Scgn is detected in the sciatic nerve and is transported peripherally from the cell body, as shown by the accumulation of Scgn-LI proximal to a compression of the sciatic nerve. Therefore, Scgn may have a function(s) not only in cell bodies but possibly also in central and peripheral nerve terminals.

A similar situation may exist for the other three CaBPs discussed here, since there is a loss of CaBPs in the ipsilateral dorsal column/column nucleus after unilateral, multiple dorsal root ganglionectomy [40] and dorsal rhizotomy [34]. The latter is the projection area of large DRG neurons [62], and this is the category of DRG neurons that to large extent harbor CR and PV.

### Other CaBPs in DRGs

CaBPs have in mouse mainly been studied as markers for the diverse neuron populations, especially during development and in cultures (e.g. [19,20,28,30-32]), but detailed *in vivo* quantitative and colocalization analyses of adult mDRGs are less common. Nevertheless, Ichikawa et al. [50] reported the presence of PV-LI in ~5% of adult mDRG NPs.

In contrast, a large number of studies have dealt with this issue in rat (for refs. see Introduction). In the most recent study on rDRGs by Ichikawa and colleagues [44] and using triple-label immunostainings, CR and PV are both present in mostly large-sized NPs, and ~10% of the NPs contain both CaBPs (most CR neurons contain PV and ~40% of PV neurons contain CR). A bimodal size curve has been earlier described for CR by the same group [39]. Neither PV nor CR coexists with CB, the latter being expressed in neurons of various sizes.

With regard to nociceptors, known to be small-sized DRG neurons [51,52], none of these three CaBP populations, mostly encompassing large neurons, seem to be extensively involved: 1% of the PV-positive NPs express CGRP [42], colocalization of CGRP with either PV or CB is “rare” [37,38]. Nevertheless, Honda [49] reported that 9% of CB neurons are CGRP-IR, and 7% of CR neurons are substance P-positive [39]. The present results suggest that Scgn is the major CaBP in a population of peptidergic nociceptors in mDRGs [51,52]. Similar to mDRGs, Scgn is also expressed in mTGs, and majority of them are CGRP-positive.

### Phylogenetic differences

We detected both similarities and differences in expression of Scgn-LI when comparing DRGs, TGs and spinal cord of mouse with rat and human tissues, suggesting partially conserved protein expression. In rDRGs, even fewer NPs were Scgn-IR as compared to mouse and, surprisingly, none of them was CGRP-IR. In contrast, Scgn expression was quantitatively similar in mouse and human DRGs, most of them expressing CGRP and none seemed IB4-positive. In rTGs many Scgn-IR neurons coexpressed CGRP and many, unexpectedly, stained for IB4, whereas no Scgn-IB4 containing neurons were detected in mouse TGs.

With regard to spinal cord, Scgn in mouse is present mainly in cell bodies in inner lamina II and, albeit in low numbers, in several other layers (I, II-IV) of the dorsal horn, and in ventral horn neurons. This pattern was similar in human spinal cord. However, cell bodies were only detected in inner lamina II, and they did not form a distinct band as in mouse. In rat, Scgn staining was less pronounced with little fiber staining in the very superficial region, possibly reflecting the low numbers of Scgn-IR cell bodies in DRGs and with the staining in inner lamina II mainly located medially. In spinal ventral horn, no Scgn-IR neurons could be detected in rat. Scgn-LI in mouse was found together with CB-, PV- or CR-LI, albeit at very different proportions. In the rat spinal cord some Scgn-IR interneurons also expressed CB or PV, but not CR-LI.

Taken together, mouse is similar to human with respect to Scgn expression in DRGs, and to large extent in the spinal cord, while rats exhibit substantial differences. With regard to CaBPs, in rDRGs more than 40% of the Scgn-IR NPs co-expressed CB, but hardly any CR or PV, a species difference here is being the low CB-Scgn coexistence in mouse.

### Scgn and other CaBPs in spinal cord

Several studies on CaBPs in the rat spinal cord have been published [21,34-36,40,48,63] but only few on mouse [29], the latter focusing on a select neuron population, so called V1 neurons in the deeper layers. The studies on rat have shown that in lamina I many neurons are CB-IR, fewer CR-IR and none PV-IR. In lamina II CB- and CR-IR neurons are densely packed, and PV is confined to a distinct band in inner lamina II. There is only limited coexistence of the three CaBPs in the superficial laminae, although examples of cell positive for both CR and CB have been observed [40]. Laminae III and IV have in general cell bodies expressing CaBPs [35,48]. In the present study Scgn was, in contrast to CB, CR and PV, also expressed in mouse ventral horn neurons.

### Functional aspects on Scgn

In view of their function as ‘gate keepers’ of Ca^2+^ homeostasis, CaBPs have been hypothesized a protective role for preventing abnormal, cytotoxic Ca^2+^ levels, thus likely participating in neurodegenerative processes and disease [64-72]. Interestingly, involvement of Scgn in neuronal survival in Alzheimer’s disease has also been reported [10-12]. There is functional evidence that Ca^2+^ buffering is important also in sensory neurons [73] and its dysfunction facilitates sensory neuron degeneration [74].

An alternative function as Ca^2+^ sensor may be considered for Scgn in DRG neurons, since an interaction between Scgn and SNAP-25 proteins has been reported by Rogstam et al. [17]. This finding suggests a role in the control of neurotransmitter release since N-ethylmaleimide-sensitive factor-attachment protein receptors (SNAREs)-associated proteins are part of the exocytotic machinery [18]. Interestingly, inhibition of exocytosis causes long-lasting attenuation of pain [75].

## Conclusions

Scgn represents a novel CaBP, which here and earlier has been found expressed in subpopulations of neurons in the rodent and primate nervous system, complementary to several other well-known members of this proteins superfamily. However, Scgn-positive DRG neurons, presumably a subtype of nociceptors both in mouse and humans, do not seem to perfectly match any of the DRG neuron populations identified during development in mice [19,20]. Scgn is also present in cell bodies in various layers of the dorsal horn. Analysis of corresponding tissues in rat suggests species differences in Scgn expression. The similarity between mouse and human DRGs suggest that results from future experiments on Scgn, e.g. using gentically modified mice, may be relevant to decipher molecular pathomechanisms in humans.

## Methods

### Tissues and animal models

Experiments were performed on male C57BL/6J Bommince mice (A/S Bomholtgaard, Ry, Denmark) weighing 25–28 g, and on adult male Sprague–Dawley rats (200–250 g; B and K Universal, Stockholm, Sweden). All animals were kept under standard conditions on a 12-hour daylight cycle with free access to food and water. Unilateral sciatic nerve transection (axotomy) was made on n=10 mice as described earlier [76]. Surgical procedures were performed under anesthesia with isoflurane. Operated animals were allowed to survive for 2 weeks after surgery. Dorsal root rhizotomy was done on n=5 mice, and animals were allowed to survive for 10 days. Sciatic nerve ligation was done in n=5 mice, which were sacrificed after 10 hours. Human ganglia were harvested from children with obstetric brachial plexus lesions, and undergoing reconstructive nerve surgery. Human spinal cord was harvested from a 48-year-old women died from stroke. The studies have been approved by the local Ethical Committee for animal experiments (Norra Stockholms djursförsöksetiska nämnd), and experiments on hDRGs were approved by a local Ethical Committee with written consent from the next of kin.

### mRNA detection in tissues

*In situ* hybridization analysis of Scgn mRNA in mouse olfactory bulbs using riboprobes was performed as previously described [77]. Briefly, adult brains were perfusion fixed followed by post-fixation in the same fixative overnight (4% paraformaldehyde, in 0.1M PB), cryoprotected (30% sucrose in 0.1M PB), embedded in Tissue-tek OCT compound (Miles Laboratories, Elkhart, IN) and sectioned at a thickness of 20 μm. The fragment of scgn cDNA used for riboprobe synthesis was amplified from an adult mouse olfactory bulb cDNA preparation by PCR using Pfu DNA polymerase (Promega). The primers used for scgn cDNA amplification were flanked at their 5’ ends with T7 and SP6 polymerase acceptors 5’-[CTGTAATACGACTCACTATAGGG] TCTCTAAGGAAGGCCGCATA -3’ (sense) and 5’- [GGGATTTAGGTGACACTATAGA] AGACACAGTGCCAGCTCAGA -3’ (antisense). Resulting amplicons were directly used as templates for *in vitro* transcription. Amplicon size was confirmed by loading PCR products onto 1% agarose gels. Digoxigenin-labeled antisense and sense riboprobes for mouse scgn were synthesized by *in vitro* transcription using SP6 and T7 RNA polymerases (Roche). After synthesis, probes were cleaned by using the RNeasy kit (Qiagen) and DNA digested with RNase-free DNase I (Qiagen). Alkaline phosphatase-conjugated anti-digoxigenin antibodies (Roche, 1:2,000) were used with their signal developed by 5-bromo-4-chloro-3-indolyl-phosphate/nitro blue tetrazolium as substrate (BCIP/NBT; Roche).

*In situ* hybridization using oligoprobes was performed as described previously [78]. Briefly, a mixture of two commercially acquired oligonucleotide probes (CyberGene, Stockholm, Sweden, or MWG Biotech, Ebersberg, Germany) were used: 1) GGACAGGCAGAGCCATCTAACAGGGGAG, 2)ACACAGGGGCTTTCAGTGAGACAGGGATAGAT complementary to nucleotide sequences of the human Scgn (accession number NM_006998.3). hDRGs were air-dried and incubated with a hybridization solution containing 0.5 ng of labeled probe/slide. The hybridization solution contained 50% deionized formamide (J.T. Baker Chemicals, Deventer, The Netherlands), 4×SSC (0.6 M sodium chloride, 0.06 M sodium citrate), 1× Denhardt’s solution (0.02% bovine serum albumin, 0.02% Ficoll (Pharmacia, Uppsala, Sweden), 0.02% polyvinylpyrrolidone), 1% N-lauroylsarcosine, 0.02 MNaPO4 (pH 7.0), 10% dextran sulfate (Pharmacia), 500 μg/ml denatured salmon testis DNA (Sigma, St. Louis, MO, USA) and 200 mM dithiothreitol (LKB, Stockholm, Sweden). Sixteen hours after incubation the tissues were rinsed in 1×SSC for 4 times (each time 15 minutes) at 56°C and allowed to cool to RT, washed in distilled water, and transferred rapidly through 60% and 95% ethanol. The 33 P-dATP-labeled sections were apposed to β-max autoradiography film (Amersham). The films were exposed for one and half months and developed with Kodak LX 24 and fixed with Kodak AL4. The slides were rinsed in distilled water and coverslipped with glycerol. The sections were then counterstained with cresyl violet, dehydrated in graded series of ethanol, and coverslipped with Entellan (Merck, Darmstadt, Germany). All sections were examined in a Nikon Microphot microscope equipped for bright- and dark-field microscopy. Photographs were taken with a Nikon Coolpix 5000 digital camera (Nikon, Tokyo, Japan).

### mRNA detection by quantitative real-time PCR

Quantitative PCR (qPCR) reactions were performed with custom designed primers according to published protocols [16]. RNA isolated from tissues microdissected from adult C57Bl/6N mice (*n* = 3/DRG/spinal cord/olfactory bulb) were subjected to Scgn expression analysis after validating RNA integrity (data not shown). Gapdh was used to normalize Scgn mRNA expression levels. Results from qPCR experiments were subsequently compared to Scgn mRNA distribution as determined by *in situ* hybridization (primers: 5’CCCAGAAGTGGATGGATTTG 3’; reverse: 5’GTTGGGGATCAGGGGTTTAT 3’.

### Immunohistochemistry

All operated (n=20) and control mice (n=10), as well as rats (n=10) were deeply anesthetized with sodium pentobarbital (10 mg/kg for mouse and 50 mg/kg for rat, both i.p.) and transcardially perfused with 20 ml (50 ml) of warm saline (0.9%; 37°C), followed by 20 ml (50 ml) of a warm mixture of 4% paraformaldehyde (37°C) and 0.4% picric acid in 0.16 M phosphate buffer (pH 7.2), and then by 50 ml (250 ml) of the same, but ice-cold fixative [79,80]. The L 5 mDRGs, mTGs, rDRGs and rTGs, as well as the L4 and L5 segments of both mouse and rat spinal cord were dissected out and postfixed in the same fixative for 90 min at 4°C. Specimens were subsequently stored in 10% sucrose in phosphate buffered saline (PBS, 0.1 M, pH 7.4) containing 0.01% sodium azide (Sigma, St. Louis, MO) and 0.02% bacitracin (Sigma) as preservatives at 4°C for 2 days. The hDRGs and spinal cord were immersion-fixed for four hours in ice-cold fixative and rinsed as mentioned above. Tissues were embedded with OCT compound (Tissue Tek), frozen and cut in a cryostat (Microm, Heidelberg, Germany) at 10 μm (mDRGs), 14 μm (mTGs, rDRGs and hDRGs) or 20 μm (mouse, rat and human spinal cords) thickness and mounted onto chrome-alum-gelatin-coated slides. Thaw-mounted sections were dried at room temperature (RT) for 30 min and rinsed with PBS for 15 min. Sections were incubated for 18 hours at 4°C in a humid chamber with rabbit anti-Scgn antiserum (1:1,000); [15,16,60] diluted in PBS containing 0.2% (w/v) bovine serum albumin and 0.03% Triton X-100 (Sigma). In addition a monospecific polyclonal rabbit anti-human Scgn antibody was used [8]. Briefly, purified recombinant Scgn (540 μg) was emulsified in complete Freund's adjuvant and injected subcutaneously into a rabbit followed by two more injections of incomplete Freund's adjuvant. Serum collected one month after the third immunization contained high titer antibody activity against the recombinant protein when tested by ELISA [8].

Immunoreactivity was visualized using the tyramide signal amplification system (TSA Plus; NEN Life Science Products, Boston, MA). Briefly, the slides were rinsed with TNT buffer (0.1M Tris–HCl, pH 7.5; 0.15 M NaCl; 0.05% Tween 20) for 15 min at RT, blocked with TNB buffer (0.1M Tris–HCl; pH 7.5; 0.15M NaCl; 0.5% DuPont blocking reagent) for 30 min at RT followed by a 30-min incubation with horseradish peroxidase-labeled swine anti-rabbit antibody (1:200; Dako, Copenhagen, Denmark) diluted in TNB buffer. After a quick wash (15 min) in TNT buffer, all sections were exposed to biotinyl tyramide-fluorescein (1:100) diluted in amplification diluent for approximately 15 min, and finally washed in TNT buffer for 30 min at RT.

For double-staining experiments, immunohistochemistry was carried out with cocktails of primary antibodies: Scgn (1:1,000) plus CGRP (1:10,000) [81], PV (1:400), CB (1:400), CR (1:400), TRPV1(1:500) (St. Cruz Biotechnology, St. Cruz, CA), GRP (1:500) (ImmunoStar, Hudson, WI) or PKCgamma (1:1,000) (St. Cruz Biotechnology), respectively, following previous published protocols [60] (the three antibodies to CaBPs were from Swant, Bellinzona, Switzerland). For triple stainings, Scgn (1:1,000) plus PV (1:400) and CB (1:400) or Scgn (1:1,000) plus CB (1:400) and CR (1:400). In addition, a group of Scgn-labeled sections was incubated with the IB4 from Griffonia simplicifolia I (GSA I; IB4; 2.5 μg/ml; Vector Laboratories, Burlingame, CA) [82] followed by incubation with a goat anti-GSA I antiserum (1:4,000; Vector Laboratories) and rhodamine red X-conjugated donkey anti-goat antibody (1:200; Jackson ImmunoResearch, West Grove, PA). hDRG slides were only processed for Scgn and CGRP or IB4, respectively. Finally, all slides were coverslipped with glycerol/PBS (9:1) containing 2.5% DABCO (Sigma) [83,84].

The specificity of Scgn antiserum was tested by preabsorption of the antiserum with homologous antigen at a concentration of 1 and/or 10 μM for 24 hours at 4°C. After incubation with control serum, i.e. Scgn antiserum pre-absorbed with the excess of Scgn, no fluorescent neuronal cells could be observed (data not shown).

### Western blot analysis

L4 and L5 mDRGs from mice with unilateral sciatic nerve transection (n=5) and controls (n=5) were removed and immediately put on dry ice. DRGs and spinal cord (L4-5 segments) were homogenized in lysis buffer (50 mM Tris–HCl, pH 7.4, 1% NP-40, 0.25% sodium deoxycholate, 150 mM NaCl, 1 mM EDTA, 1 mM NaF and 1 mM Na3VO4) containing protease inhibitor cocktail (Sigma) using sonication. Lysates were centrifuged at 12,000 rpm for 20 min at 4°C. The supernatant was collected for western blots. Protein concentration was measured by Bradford’s Assay (Bio-Rad, Hercules, CA). Laemmeli sample buffer containing about 20 μg of protein was loaded in each lane and separated on 10% SDS-PAGE gel, then transferred to polyvinylidene fluoride (PVDF) membranes (Millipore, Hemel, Hempstead, UK). The membranes were blocked with 5% non-fat dry milk in PBS with 0.1% Tween-20 for 1 hour at RT and incubated overnight at 4°C with an antibody against Scgn (for DRG sample, rabbit polyclonal, 1:1,000; for spinal cord sample, mouse monoclonal, 1:1,000; Atlas antibody clone 13B8). The membranes were incubated with HRP-conjugated secondary antibodies for 1 hour at RT (1: 5,000-1: 10,000, DAKO) followed by ECL solution for 5 min (Amersham Biosciences, Piscataway, NJ) and exposed to X-ray film (NEN PerkinElmer, Waltham, MA). The membrane was stripped and re-probed for β-Actin (mouse monoclonal, 1: 5,000-10,000, Cell Signaling Technology, Danvers, MA) as loading control. Images were quantified with Quantity One software on non-saturated images (Bio-Rad).

### Image analysis and quantification

Specimens were analyzed on a Bio-Rad Radiance Plus confocal scanning microscope (Bio-Rad, Hemel, Hempstead, UK) installed on a Nikon Eclipse E 600 fluorescence microscope (Nikon, Tokyo, Japan) equipped with x10 (0.5 numerical aperture, NA), x20 (0.75 NA) and x60 oil (1.40 NA) objectives. Fluorescein labeling was excited using the 488-nm line of the argon ion laser and detected after passing a HQ 530/60 (Bio-Rad) emission filter. For the detection of lissamine rhodamine sulfonyl chloride and rhodamine, the 543-nm HeNe laser was used in combination with the HQ 570 (Bio-Rad) emission filter. For the detection of DAPI a 405-nm laser was used. All the slides were scanned in a series of 1μm-thick optical sections. Consequently, images were analyzed separately and merged to evaluate possible colocalization. In some cases a Zeiss laser scanning microscope 780 system with a plan-apochromat x 20 (0.8NA) M27 objective was used.

To determine the percentage of IR NPs, the counting was performed on 10 or 14 μm thick sections, and every 4th or 6th section was selected (Nike Microphot-FX microscope, 20x objectives). The total number of immuno-positive NPs was divided by the total number of propidium iodide-stained [85] NPs in the DRG sections, and the percentage of positive NPs was calculated. Five to ten sections of each DRG from five animals in each group were included in the analysis, and 1,200-3,000 NPs were counted in each ganglion. The size distribution of NPs with a visible nucleus was measured using the Nikon Eclipse E 600 fluorescence microscope with Wasabi Image Software. We divided the NPs into small (a somal area of 100–600 μm^2^); medium-sized (600–1400 μm^2^) and large (>1400 μm^2^) according to earlier studies [76,86]. The percentages of DRG NPs in each of these categories were calculated.

### Statistical analyses

Differences between the percentage of Scgn-IR NPs as well as the gray levels of Scgn in mDRG neurons in ipsilateral and contralateral samples were evaluated by Student’s *t* test.

## Abbreviations

CaBP: Calcium-binding protein; CB: Calbindin D-28k; CGRP: Calcitonin gene-related peptide; CR: Calretinin; GRP: Gastrin releasing peptide; m/r/hDRG: Mouse/rat/human dorsal root ganglion; IB4: Isolectin B4; IR: Immunoreactive; L: Lumbar; LI: Like immunoreactivity; NP: Neuron profile; PKCgamma: Protein kinase C gamma; PV: Parvalbumin; RT: Room temperature; Scgn: Secretagogin; TG: Trigeminal ganglion; TRPV1: Transient receptor potential vanilloid subtype 1.

## Competing interests

The authors declare no competing interests.

## Authors’ contributions

TJSS and ToH designed research; TJSS, QX, MDZ and GT performed research; HH, JM, LW, MU, AJ, KF and TiH contributed new reagents/analytical tools; TJSS, QX, MDZ and GT analyzed data; TJSS, QX, MDZ, JM, TiH and ToH wrote the paper. All authors read and approved the final manuscript.

## Supplementary Material

Additional file 1 **Figure S1.** (A) Overview of *in situ* hybridization to detect Scgn mRNA in the adult mouse olfactory bulb using a riboprobe. Open rectangles indicate the locations of (A1,A2). (A1) Cellular Scgn mRNA detection (arrowheads) in the external plexiform layer (ML: mitral cells are negative). (A2) Hybridization signal, although at markedly lower levels, is also seen in periglomerular cells (arrowheads). (B) Sense control showing the lack of non-specific signal detection/amplification. (C) Immunohistochemistry for Scgn (red) in the mouse olfactory bulb. Note that layer-specific cellular immunoreactivity confirms mRNA distribution. CR (blue) is used to identify the glomerular layer. (C1) High-resolution image of neurons at locations corresponding to that in (A1, arrowheads). Abbreviations: EPL, external plexiform layer; GL, glomerular layer; GRL, granular layer; ML, mitral layer Scale bars = 200 um (A,B), 100 um (C), 10 um (A1,A2,C1). Click here for file
