# Supplementary figures and images for: Secretagogin is expressed in sensory CGRP neurons and in spinal cord of mouse and complements other calcium-binding proteins, with a note on rat and human
Source: Mol Pain. 2012 Oct 29;8:80. doi: 10.1186/1744-8069-8-80 (PMC3560279; doi:10.1186/1744-8069-8-80)

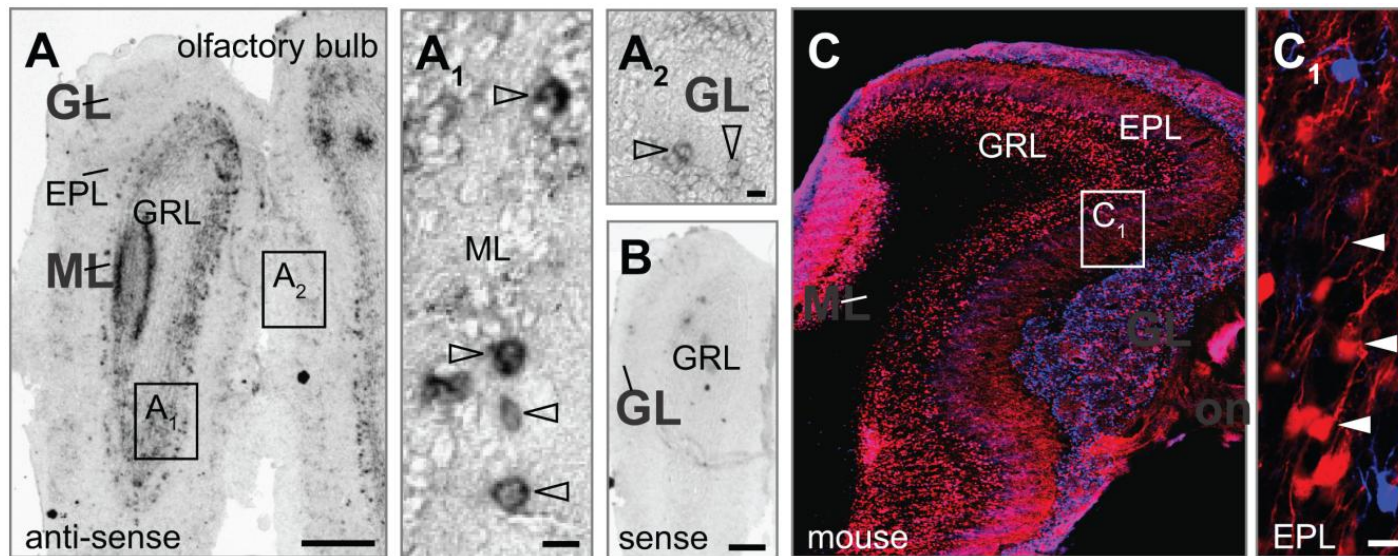

Supplement: Additional file 1 — Figure S1. (A) Overview of in situ hybridization to detect Scgn mRNA in the adult mouse olfactory bulb using a riboprobe. Open rectangles indicate the locations of (A1,A2). (A1) Cellular Scgn mRNA detection (arrowheads) in the external plexiform layer (ML: mitral cells are negative). (A2) Hybridization signal, although at markedly lower levels, is also seen in periglomerular cells (arrowheads). (B) Sense control showing the lack of non-specific signal detection/amplification. (C) Immunohistochemistry for Scgn (red) in the mouse olfactory bulb. Note that layer-specific cellular immunoreactivity confirms mRNA distribution. CR (blue) is used to identify the glomerular layer. (C1) High-resolution image of neurons at locations corresponding to that in (A1, arrowheads). Abbreviations: EPL, external plexiform layer; GL, glomerular layer; GRL, granular layer; ML, mitral layer Scale bars = 200 um (A,B), 100 um (C), 10 um (A1,A2,C1). [file 1744-8069-8-80-S1.pdf]
